# Efficacy and Safety of Shengmai Injection for Chronic Heart Failure: A Systematic Review of Randomized Controlled Trials

**DOI:** 10.1155/2020/9571627

**Published:** 2020-06-20

**Authors:** Yanping Wang, Xu Zhou, Xiaofan Chen, Fei Wang, Weifeng Zhu, Dongmei Yan, Hongcai Shang

**Affiliations:** ^1^Evidence-based Medicine Research Center, Jiangxi University of Traditional Chinese Medicine, Nanchang, Jiangxi, China; ^2^Key Laboratory of Chinese Internal Medicine of Education and Beijing, Dongzhimen Hospital, Beijing University of Chinese Medicine, Beijing, China

## Abstract

**Background:**

Shengmai injection (SMI) is made from purified ginseng, Radix Ophiopogonis, and *Schisandra chinensis*. It has cardiotonic effects and is clinically used for the adjuvant treatment of chronic heart failure (CHF). However, its efficacy and safety are uncertain. The purpose of this study was to systematically evaluate the existing efficacy and safety evidence in randomized controlled trials (RCTs) that studied SMI for the treatment of CHF.

**Methods:**

PubMed, Embase, Cochrane Library, clinicaltrials.gov, CNKI, Wanfang, VIP, and CBM databases were searched up to September 10, 2019. RCTs that compared basic Western medicine treatment with SMI + basic Western medicine were included. The Cochrane Collaboration Risk of Bias Tool was used to assess the risk of bias in the RCTs. The meta-analysis used the random effects model; the mean difference (MD) and 95% confidence intervals (CIs) were combined using the inverse variance method, and the Mantel–Haenszel method was used to combine the relative risk (RR) and 95% CIs. Heterogeneity was assessed using I^2^ and *Q* tests, and the source of heterogeneity was explored by analyzing three preset subgroup hypotheses.

**Results:**

A total of 20 RCTs were included (*n* = 1562), with a moderate-to-high risk of bias. The meta-analysis showed that, compared with Western medicine alone, SMI adjuvant therapy significantly improved cardiac function indicators, including left ventricular ejection fraction (MD 6.8%, 95% CI 4.68 to 8.91), stroke volume (MD 9.81 ml, 95% CI 5.67 to 13.96), cardiac output (MD 0.96 L/min, 95% CI 0.66 to 1.25), and cardiac index (MD 0.53 L/min, 95% CI 0.36 to 0.70); heterogeneity was generally high among these outcomes. Compared with the controls, patients receiving SMI adjuvant therapy also had a higher response to treatment (RR 2.89, 95% CI 2.10 to 3.99; I^2^ = 0%), a greater decrease in brain natriuretic peptide levels (MD −284.66 ng/l, 95% CI −353.73 to −215.59, I^2^ = 0%), and a greater increase in six‐minute walk test performance (MD 70.67 m, 95% CI 22.92 to 118.42; I^2^ = 84%). Nine studies reported mild adverse events, such as gastrointestinal reactions, and no serious adverse events were reported.

**Conclusion:**

Currently, available evidence indicates that SMI, as an adjuvant for basic Western medicine treatment, can improve the cardiac function of patients with CHF with good safety outcomes. Because of the high risk of bias among the included RCTs and the large heterogeneity of partial outcomes, the findings of this study must be verified by high-quality studies with large sample sizes.

## 1. Introduction

Chronic heart failure (CHF) is a complex clinical syndrome [1] and a common outcome of acute and chronic myocardial injuries, such as coronary heart disease and rheumatic heart disease [2]. CHF has become a major issue threatening human health, with more than 23 million individuals affected worldwide [3]. In 2014, the CHF prevalence rate reached approximately 1.7% (5.7 million) and 0.3% (4.5 million) with 870,000 and 500,000 new cases in the United States and China, respectively [4, 5]. Each year, 8–10 million patients are hospitalized due to CHF in the United States [6]. As the world population continues to age, high morbidity, disability, and mortality rates can be forecasted for CHF in the future [7–9].

Currently, the focus of CHF treatment is to inhibit myocardial remodeling and restore cardiac function [10]. Conventional drug treatments include diuretics, cardiotonic steroids, angiotensin-converting enzyme inhibitors (ACEIs), and so on. Although these drugs can relieve symptoms and prevent acute attacks to some extent, an improvement in efficacy is still desirable. In China, traditional Chinese medicine (TCM) can be used to treat CHF, and it has been widely used as a complementary therapy in combination with Western medicine [11, 12].

According to TCM theory, CHF is classified into the categories of heart palpitations, dyspnea, and phlegm-retained fluid. Its etiology is the deficiency of Qi and blood, blood stasis, and phlegm-dampness, and its pathogenesis is characterized by deficient root and excessive superficiality [13]. Therefore, replenishing Qi and nourishing Yin are the main treatment methods. With the modernization of TCM, a group of compound TCM injections prepared with purified TCM formulas for the treatment of CHF has been invented in China [14]; these compounds are administered directly into the body through intravenous injection and therefore have a fast onset and high bioavailability [15].

Shengmai injection (SMI), which is composed of ginseng, Radix Ophiopogonis, and Schisandra chinensis, is a typical TCM injection for the treatment of CHF. SMI originated from a classic TCM formula, Sheng-Mai San, which was first recorded during the Jin Dynasty [16]. According to TCM theory, Sheng-Mai San has the effects of replenishing Qi and nourishing Yin and thus is beneficial for patients with CHF, which is supported by its efficacy for over 800 years of empirical application [17]. SMI is made by using modern methods to extract the active ingredients of Sheng-Mai San, which theoretically acts faster and has better efficacy. Studies also have shown that SMI can reduce cardiac mass and left ventricular mass in CHF rats, indicating a beneficial effect on ventricular remodeling and cardiac function [18]. In fact, SMI has been widely used in clinical practice as an adjuvant treatment for CHF in China [19]. However, because of China's early drug marketing policy, many TCM injections, including SMI, lack rigorous efficacy and safety evaluations.

Currently, some randomized controlled trials (RCTs) have investigated the effects of SMI on CHF, but most have small sample sizes, and the results are not consistent. For example, some RCTs [20, 21] indicated a higher response rate and greater improvements in cardiac function indicators after SMI adjuvant therapy compared with Western medicine alone therapy, while others did not show this relationship [22]. Additionally, adverse events were reported in some RCTs [23], such as nausea, bloating, and rash, but their association with SMI remains unclear [24]. Therefore, we conducted a systematic review of existing RCT evidence to assess the efficacy and safety of CHF for SMI in detail and to provide integrated evidence for the clinical use of this drug.

## 2. Methods

We compiled the results according to the Preferred Reporting Items for Systematic reviews and Meta-Analyses statement (PRISMA) [25].

### 2.1. Data Sources and Searches

We searched eight databases (PubMed, Embase, Cochrane Library, clinicaltrials.gov, CNKI, Wanfang, VIP, and CBM) from their inception through to September 10, 2019, without language restrictions. Keywords and medical subject heading terms were used to construct a search strategy. For example, the search strategy in PubMed was “heart failure [mh] OR chronic heart failure ∗ [tiab] OR cardiac failure ∗ [tiab] OR heart failure ∗ [tiab] OR congestive heart failure ∗ [tiab] OR diastolic heart failures ∗ [tiab] OR systolic heart failures ∗ [tiab] OR cardiac decompensation ∗ [tiab] OR heart decompensation ∗ [tiab]) AND (shengmai injection ∗ [tiab] OR sheng mai injection ∗ [tiab]) NOT (animals[mh] NOT humans[mh])”. The search strategies for the other databases were adjusted based on their search rules.

### 2.2. Eligibility Criteria

Eligible studies were those that met all of the following criteria: (1) the study was designed as an RCT with a description of the randomization method; (2) patients were diagnosed with CHF by the Framingham criteria [26] and their cardiac function was graded as two to four by the American Heart Association criteria [27]; (3) SMI was assessed as an adjuvant therapy to basic Western medicine, and there were no restrictions on the dose and course of treatment; (4) basic Western medicine (e.g., diuretics, angiotensin receptor blockers, ACEIs) was used as a comparator; and (5) data of at least one outcome of interest, including left ventricular ejection fraction (LVEF), response to treatment assessed by the New York Heart Association (NYHA) standards [27], stroke volume, cardiac output, cardiac index, brain natriuretic peptide (BNP), six-minute walk test (6MWT), and adverse events, were available. The NYHA standards classify the cardiac function into four grades, and the response to treatment is classified as follows: (1) marked response: cardiac function displays an improvement of ≥2 grades or reaches grade one; (2) moderate response: the cardiac function displays an improvement of one grade; (3) no response: no improvement of cardiac function. Studies that assessed other forms of SMI, such as capsules or granules, were excluded, as were studies in which SMI was combined with other TCMs.

### 2.3. Screening of Studies and Data Extraction

The bibliographies obtained in the search were imported into Endnote for deduplication and then screened and cross-checked by two reviewers. Titles and abstracts were read for preliminary screening of irrelevant studies, and the full papers were read to determine whether they were relevant for inclusion. If there was a disagreement between the two reviewers, a third researcher made the final decision. The following information was extracted for each included study: first author; year; sample size; gender; age; course of CHF; cardiac function grade; type; dose and course of treatment and control; length of follow-up; outcome data.

### 2.4. Risk of Bias in Individual Studies

The risk of bias of the included RCTs was assessed using the Cochrane 5.1.0 assessment tool [28], which includes the following seven items: (1) adequate random number generation; (2) adequate allocation concealment; (3) adequate blinding of patients and doctors; (4) adequate blinding of outcome assessors; (5) free from infrequent missing outcome data; (6) free from selective outcome reporting; (7) free from other sources of bias. Each item was judged as “low risk,” “high risk,” or “uncertain.” We finally judged an overall high study-level risk of bias when the answer was “high risk” for any item and an overall low study-level risk of bias when the answers were “low risk” for all items; the rest of the studies were judged as a moderate study-level risk. Two reviewers independently evaluated the risk of bias and cross-checked the results. Any discrepancy was resolved by a third reviewer.

### 2.5. Statistical Synthesis and Analysis

Data from individual RCTs were combined in the meta-analysis using the random effects model. The mean difference (MD) and its 95% confidence intervals (CIs) were used as the effect measures for continuous variables and combined by the inverse variance method. The Mantel-Haenszel method was used to combine the relative risk (RR) and 95% CIs for dichotomous variables. *I*^2^ and *Q* tests were used to evaluate heterogeneity where statistically significant heterogeneity was defined as *I*^2^ >50% or *P* < 0.10 in the *Q* test. The source of heterogeneity was explored by analyzing the variables in three preset subgroups, including treatment course (three weeks as the cut-off point), SMI dose (60 ml/day as the cut-off point), and mean patient age in the SMI group (65 years as the cut-off point). The stability of the results was examined by a sensitivity analysis excluding studies with a high study-level risk of bias. For outcomes in which 10 or more RCTs were analyzed, an inverted funnel plot was used to evaluate publication bias. The meta-analyses were performed using RevMan v5.3 (St. Louis, MO, USA).

## 3. Results

### 3.1. Description of Studies

**Figure 1:**
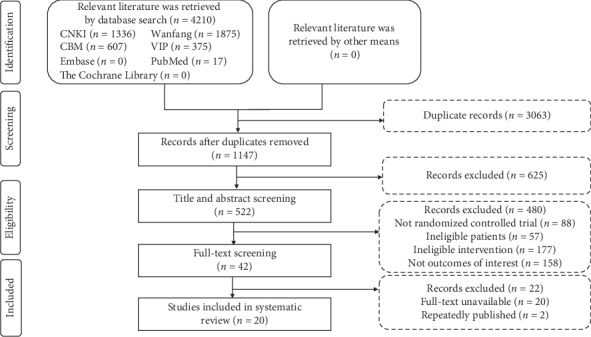
Flowchart of study selection.

A total of 4210 papers were retrieved, and 20 RCTs involving 1562 patients with CHF [22, 23, 29–46] were eventually included (Figure 1). In the included RCTs, the sample sizes ranged from 50 to 178, of which 62.3% of patients were males. Patients with grades two, three, and four cardiac function accounted for 23.4%, 52.9%, and 23.7% of the study populations, respectively. The SMI dose ranged from 30 to 100 ml/day, with a treatment course of one to four weeks. There were no significant differences in the baseline characteristics between the SMI and control groups in the individual RCTs (Table 1).

### 3.2. Risk of Bias

**Figure 2:**
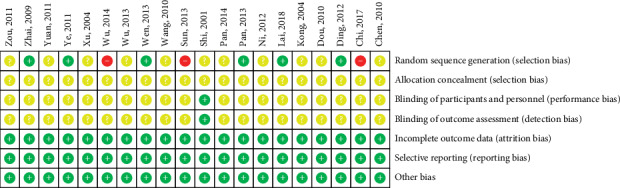
Risk of bias summary.

For the methods used to generate random numbers, six out of 20 RCTs [22, 23, 34, 39, 43, 45] used the random number table (low risk of bias), three [30, 37, 40] were based on admission number (high risk of bias), and the rest were unclear. None of the RCTs specified the methods of allocation concealment. One RCT [35] blinded patients, doctors, and outcome evaluators, whereas the rest had no information related to the methods of blinding. No patients were lost during the follow-up. There was no evidence of the possibility of selective reporting and other sources of risk of bias. Overall, three RCTs were rated as having a high risk of bias, and the rest had a moderate risk of bias (Figure 2).

### 3.3. LVEF

**Figure 3:**
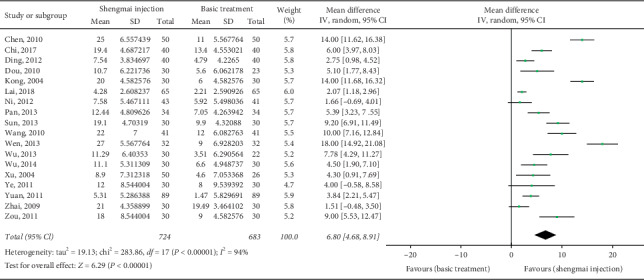
Meta-analysis of data on left ventricular ejection fraction (%).

Eighteen RCTs (*n* = 1407) [22, 23, 29–34, 36–47] reported LVEF data before and after treatment. As shown in Figure 3, the meta-analysis showed that the improvement in LVEF after SMI adjuvant treatment was significantly better than that after Western medicine treatment alone (MD 6.8%, 95% CI 4.68 to 8.91, *P* < 0.00001). The heterogeneity across the primary studies was high (*I*^2^ = 94%).

### 3.4. Response to Treatment

**Figure 4:**
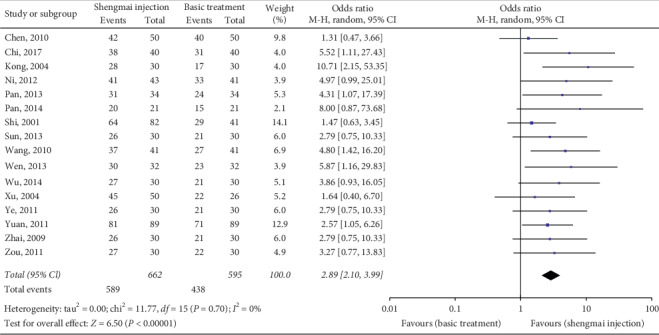
Meta-analysis of data on response to treatment.

Sixteen RCTs [29, 30, 32–40, 42–46] (*n* = 1317) reported the response to treatment. In the SMI group, 284 (41%), 333 (48%), and 75 (11%) patients had marked, moderate, and no responses, respectively; these values were 189 (30%), 266 (42.6%), and 170 (27.4%) in the control group, respectively. As shown in Figure 4, the meta-analysis was conducted after combining the marked and moderate responses to calculate the response rate. The results showed that the response rate of the SMI group was significantly higher than that of the control group (RR 2.89, 95% CI 2.10 to 3.99, *P* < 0.00001), with a low study-level heterogeneity (*I*^2^ = 0%).

### 3.5. Stroke Volume

**Figure 5:**
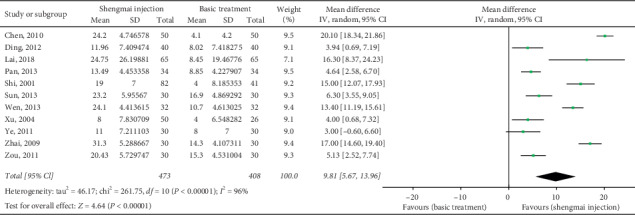
Meta-analysis of data on stroke volume (ml).

Eleven studies (*n* = 881) [22, 23, 29, 34, 36, 37, 39, 42, 43, 45, 46] reported an intergroup comparison of stroke volume. The combined results suggested that, compared to baseline, the increase in stroke volume in the SMI group was significantly greater than that in the control group (MD 9.81 ml, 95% CI 5.67 to 13.96, *P* < 0.00001; *I*^2^ = 96%; Figure 5).

### 3.6. Cardiac Output

**Figure 6:**
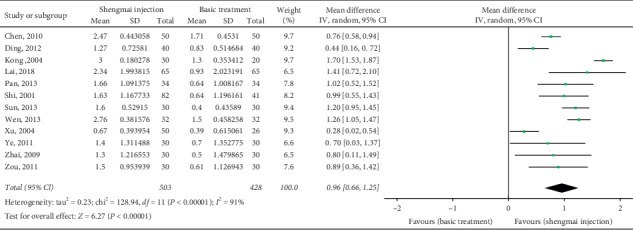
Meta-analysis of data on cardiac output (L/min).

Cardiac output was detected in 12 studies (*n* = 931) [22, 23, 29, 32, 34, 36, 37, 39, 42, 43, 45, 46]. The combined results showed that, compared with the control group, the SMI group had a significantly stronger promoting effect on cardiac output (MD 0.96 L/min, 95% CI 0.66 to 1.25, *P* < 0.00001; Figure 6). The heterogeneity was significant (*I*^2^ = 91%).

### 3.7. Cardiac Index

**Figure 7:**
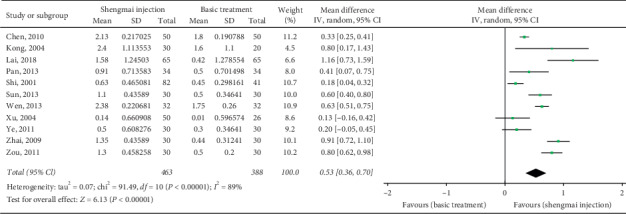
Meta-analysis of data on cardiac index (L/min).

Eleven studies (*n* = 931) [22, 29, 32, 34, 36, 37, 39, 42, 43, 45, 46] reported the cardiac index. The meta-analysis results showed that the SMI adjuvant treatment had a greater improvement in the cardiac index than the Western medicine alone; the difference was statistically significant (MD 0.53 L/min, 95% CI 0.36 to 0.70, *P* < 0.00001; *I*^2^ = 89%; Figure 7).

### 3.8. 6MWT

Three studies [22, 31, 40] reported data on changes in 6MWT, showing that the increase in 6MWT in the SMI group was greater than that in the control group (MD 70.67 m, 95% CI 22.92 to 118.42, *P*=0.004; heterogeneity: *I*^2^ = 84%).

### 3.9. BNP

BNP levels were reported in three studies [22, 33, 44]. The meta-analyses results showed that, compared with that for Western medicine treatment alone, the decrease in BNP levels was significantly greater for the combined SMI and Western medicine treatment (MD -284.66 ng/l, 95% CI -353.73 to -215.59, *P* < 0.00001; *I*^2^ = 0%).

### 3.10. Additional Analysis

#### 3.10.1. Subgroup Analysis

The subgroup analysis of different treatment courses showed that SMI treatment >3 weeks could provide significantly greater improvements in stroke volume (MD 10.40 vs. 4.00 ml; test for subgroup difference: *P*=0.02), cardiac output (MD 1.03 vs. 0.28 L/min; test for subgroup difference: *P*=0.00001), and cardiac index (0.57 vs. 0.13 L/min; test for subgroup difference: *P*=0.01) than SMI treatment < 3 weeks. However, there were no significant subgroup differences for different doses of SMI and different patient ages. The detailed results of the subgroup analyses are shown in Figures S1–S12.

#### 3.10.2. Sensitivity Analysis

Sensitivity analyses were conducted after excluding three studies with a high risk of bias [30, 37, 40]. The results suggested no directional changes on the effects of SMI for all outcomes (Table S1).

### 3.11. Publication Bias

The funnel plots (Figures S13–S17) show that the distributions of the effect sizes of LVEF and response in the studies were asymmetric, which was confirmed by Egger's test (*P*=0.016 and *P*=0.001, respectively). The funnel plots for stroke volume (Egger's test, *P*=0.244), cardiac output (Egger's test, *P*=0.437), and cardiac index (Egger's test, *P*=0.318) were symmetric, indicating that these outcomes had no significant publication bias. Fewer than 10 studies assessed 6MWT and BNP; therefore, tests for publication bias were not performed for these outcomes.

### 3.12. Safety Analysis

Nine RCTs [22, 23, 30, 33, 36, 39, 42, 43, 45] reported safety outcomes. Chi [30] reported three (7.5%) cases of nausea, two (5%) cases of bloating, and two (5%) cases of rash in the SMI group and three cases of nausea (7.5%) and two headaches (5%) in the control group. Lai et al. [22] reported one (1.5%) case of nausea, two (3%) cases of dizziness, and two (3%) cases of sinus bradycardia in the SMI group and four, six, and six cases of the above adverse events in the control group, respectively. In Shi [36], only three (7%) cases of nausea occurred in the control group, and the symptoms disappeared after the discontinuation of treatment; the SMI group had no adverse events. The remaining six RCTs reported no significant adverse events in either group.

## 4. Discussion

To our knowledge, this is the first systematic review focusing on the efficacy and safety of SMI adjuvant therapy for CHF. The meta-analyses showed that, with SMI adjuvant treatment, primary outcomes, including LVEF and response rate, and secondary outcomes all improved. However, due to the high risk of bias level, high heterogeneity of partial outcomes, and/or the possibility of publication bias of the included studies, we must carefully explain these results.

LVEF is a direct indicator reflecting the cardiac function and prognosis of patients with CHF; the lower the LVEF, the worse the prognosis [2]. Its normal reference value is 50–70%, and it can be increased by 5–15% after conventional drug treatment [7]. In this systematic review, the baseline LVEF was 38%, which increased by 12% after conventional drug treatment; an additional increase of 6.8% was observed after SMI treatment, which may have clinical significance. Ginsenoside in SMI may be the main effective constituent to explain the cardiotonic effect. Ginsenoside is a vasoactive component that can activate Na⁺/K⁺-ATPase activity, reduce apoptosis after myocardial ischemic injury, promote angiogenesis, and restore normal myocardial oxidative metabolism and energy balance, thereby increasing the myocardial contraction and improving the cardiac function [48]. Studies in a rat model showed that the mechanism underlying reduced apoptosis by SMI is related to increased Bcl-2 expression and reduced Bax and caspase3 expression and that the induction of angiogenesis is related to the increase in vascular endothelial growth factor [49]. Moreover, as an inflammatory response factor, tumor necrosis factor *α* (TNF-*α*) levels ≥1.0 ng/ml can inhibit myocardial contraction and be an indicator of heart failure severity. SMI purified ginseng and Radix Ophiopogonis have a strong cardiac glycoside-like effect and can reduce serum TNF-*α* levels, thus delaying myocardial remodeling [50].

This study showed that SMI could also significantly improve stroke volume, cardiac output, and cardiac index. Stroke volume is the volume of blood delivered by the ventricle in one heartbeat, cardiac output is the amount of blood pumped by the heart per minute, and cardiac index is the ratio of the amount of blood the heart pumps per minute to the surface area; these outcomes are positively correlated with LVEF. Therefore, the analysis of secondary outcomes can be considered as a consistency verification of the LVEF results. Because the studies providing secondary outcomes were highly heterogeneous and the subgroup analyses failed to explain most of the heterogeneous sources, the estimation accuracy might deviate. However, we noticed that, in the forest plots, for the above four outcomes, almost every individual result was on the right side of the invalid line (indicating that SMI was significantly effective), so although the estimation was not accurate enough, the estimation of the results did not mistake the direction.

This study found that, after SMI treatment, patients displayed a significant improvement in physical strength, which was supported by the increases in the response rate assessed by the NYHA functional classification system and 6MWT performance. There is a lack of pharmacological mechanism evidence explaining how SMI can enhance physical strength for patients with CHF; however, it can be explained according to TCM theory, in which the etiology of CHF is mainly considered to be Qi and Yin deficiencies [13]. In the SMI formula, ginseng is the main component that replenishes Qi, Radix Ophiopogonis primarily nourishes Yin, and *S. chinensis* is an adjuvant component for warming Yang, which can help ginseng and Radix Ophiopogonis reinforce Qi and Yin [16, 51]. The combined use of these three drugs has the synergistic effects of replenishing Qi, nourishing Yin, and replenishing fluids, and thus it is beneficial for relieving CHF. However, these mechanism explanations are only based on TCM theory and thus require future scientific studies.

The results also suggested that SMI can reduce the BNP level. BNP promotes sodium excretion, urination, and strong relaxation of blood vessels. When cardiac dysfunction occurs, the natriuretic peptide system is activated, and ventricular overload increases, resulting in increased BNP release [52]. Therefore, the inhibitory effect of SMI on BNP can be considered the indirect result from the improvement of cardiac function in patients, and it can also reflect the overall efficacy on CHF. However, the number of included BNP analyses and the sample size were both small. Although the heterogeneity between these studies was low, the accuracy may still be insufficient.

The RCTs included in our review reported that the SMI group experienced a small number of adverse events, including nausea, bloating, rashes, low blood pressure, and dizziness. Unfortunately, none of the studies reported the association between adverse reactions and SMI. According to this systematic review, all adverse events were mild, and the incidence was low; therefore, SMI appears to be safe. Nevertheless, acute and serious adverse reactions caused by TCM injections are not uncommon and attract significant attention, including those caused by SMI. In fact, a large-sample safety monitoring study [24, 53] has reported 1012 cases of adverse reactions caused by SMI, and most of those were acute. The most frequent reaction was fever with systemic damage (38.34%, 388/1012), followed by asthma (5.34%, 54/1012), and the most severe reaction was an anaphylactic shock (1.48%, 15/1012). Therefore, monitoring adverse reactions remains necessary after the clinical application of SMI, as is standardizing its rational use.

This systematic review comprehensively identified the relevant literature, developed evaluation plans, and strictly implemented those plans. These methodological advantages can improve the accuracy and clinical applicability of the results of this study. However, this study has some limitations: first, the overall risk of bias in the included studies was generally high. Although the sensitivity analysis showed no significant changes in the results after excluding the studies with a high risk of bias, the overall moderate-to-high risk of bias was still the main limitation of this study. Second, as mentioned above, although a small part of the heterogeneity was explained by the subgroup analysis, the residual heterogeneity was still large, and the estimation accuracy was likely affected. Third, there was a significant publication bias for the LVEF evaluation results; thus, its quality of evidence should be lowered accordingly. Fourth, because of the short follow-up time of the included studies, this study could not provide evidence for the long-term end points, such as whether SMI could ultimately reduce CHF mortality.

## 5. Conclusion

Current RCT evidence shows that compared with Western medicine alone, SMI adjuvant treatment for CHF can increase the LVEF and clinical effective rate and simultaneously improve stroke volume and cardiac output, with good safety outcomes. However, the risk of bias in the included studies was generally moderate to high, and the accuracy of some of the results was affected by heterogeneity. In the future, carefully designed large-sample RCTs and long follow-up observational studies should be performed to verify the efficacy and safety of SMI for treating CHF.

## Figures and Tables

**Table 1:** Characteristics of included studies.

Author	Sample (T/C)	Age (T/C, year)	Male (T/C, %)	Course of disease (year)	Cardiac function grade (T/C)			Dose of shengmai injection	Basic treatment^*∗*^	Outcomes
					II	III	IV			
Chen and Wang 2010 [29]	50/50	56.5/47.8	62/68	—	8/10	37/34	5/6	60 ml, q.d, 2 weeks	Diuretics, ACEI, *β*-blockers, CG	1, 2, 3, 4, 5
Chi 2017 [30]	40/40	63.1	57.5/67.5	9.4	17	45	18	50 ml, q.d, 4 weeks	Diuretics, ARB, *β*-blockers, CG	1, 2, 8
Ding 2012 [23]	40/40	70.7/72.6	45/37.5	8.70/8.9	—	30/31	10/9	40 ml, q.d, 1 week	Diuretics, ACEI, ARB, *β*-blockers, CG, nitroglycerin	2, 3, 4, 8
Dou 2010 [31]	30/23	66.5/67.1	60/61	8.5/7.4	10/7	16/10	4/6	100 ml, q.d, 4 weeks	ACEI, *β*-blockers, CG	2, 7
Kong and Zhu 2004 [32]	30/20	59/58	60/55	17/16	9/7	15/10	6/3	30 ml, q.d, 2 weeks	Diuretics, ACEI, CG,	1, 2, 4, 5
Lai 2018 [22]	65/65	61.6/65.1	65/68	11.3/12	24/21	25/25	16/15	100 ml, q.d, 1 week	ACEI, *β*-blockers, CG	2, 3, 4, 5, 6, 7, 8
Ni 2012 [33]	43/41	<80	—	2	—	—	—	50 ml, q.d, 2 weeks	Diuretics, ACEI, *β*-blockers, CG, nitroglycerin	1, 2, 6, 8
Pan 2013 [34]	34/34	63.5	82/88	2	32	36	—	60 ml, q.d,2 weeks	*β*-Blockers, CG, phentolamine	1, 2, 3, 4, 5
Pan 2014 [35]	21/21	59.3/56.9	90/61	2	—	—	—	50 ml, q.d, 2 weeks	Diuretics, ACEI, *β*-blockers, nitroglycerin, CG	1
Shi 2001, et al. [36]	82/41	65.6/64.5	57/56	10.2/9.7	19/9	41/20	22/12	100 ml, q.d, 2 weeks	Diuretics, CG, nitroglycerin, antiarrhythmic medicines	1, 3, 4, 5, 8
Sun 2013 [37]	30/30	61.4/60.5	63/60	5.92/5.5	—	—	—	60 ml, q.d, 2 weeks	Diuretics, ACEI, *β*-blockers, CG, isosorbide dinitrate	1, 2, 3, 4, 5
Wang 2010 [38]	41/41	63.6/64.3	63/39	5.8/6.2	14/15	21/20	6/5	40 ml, q.d, 2 weeks	Diuretics, *β*-blockers, CG	1, 2
Wen 2013 [39]	32/32	61/64	56/53	8.5/9	—	18/14	14/15	50 ml, q.d, 2 weeks	Diuretics, ACEI, *β*-blockers, CG, nitroglycerin	1, 2, 3, 4, 5, 8
Wu 2013 [40]	30/22	62/61.5	60/68	10.5/8	10/7	17/13	3/2	60 ml, q.d, 2 weeks	Diuretics, ACEI, *β*-blockers, CG	1, 2
Wu 2014 [41]	30/30	61.8/61.3	43/47	2.75	7/7	19/20	4/3	40 ml, q.d, 2 weeks	Diuretics, ACEI, ARB, *β*-blockers, CG, nitroglycerin	2, 7
Xu 2004 [42]	50/26	62/62.5	56/73	5.6/5.8	—	32/17	18/9	100 ml, q.d, 4 weeks	ACEI, CG, *β*-blockers	1, 2, 3, 4, 5, 8
Ye 2011 [43]	30/30	62/64	60/53	10/10	—	19/18	11/12	40 ml, q.d, 2 weeks	Diuretics, ACEI, *β*-blockers, CG, nitroglycerin	2, 3, 4, 5, 8
Yuan 2011 [44]	89/89	45–74	72/83	8	74	66	49	50 ml, q.d, 3 weeks	Diuretics, ACEI, *β*-blockers,	1, 2
Zhai 2009 [45]	30/30	62/63	60/53	10/10	—--	17/18	13/12	40 ml, q.d, 2 weeks	Diuretics, ACEI, *β*-blockers, nitroglycerin, CG	1, 2, 3, 4, 5, 8
Zou 2011 [46]	30/30	67.4/64.3	63/70	4.5/4.8	7/8	14/12	9/10	40 ml, q.d, 2 weeks	Diuretics, ACEI, *β*-blockers, CG	1, 2, 3, 4, 5

^*∗*^Both patients in the shengmai injection and control group received the same basic treatment in all individual randomized controlled trials. *T* = treatment; *C* = control; ACEI = angiotensin-converting enzyme inhibitors; ARB = angiotensin receptor blockers; CG = cardiac glycosides. 1: clinical efficacy; 2: left ventricular ejection fractions; 3: stroke volume; 4: cardiac output; 5: cardiac index; 6: brain natriuretic peptide; 7 : six min walk test; 8: adverse events.

## Abbreviations

SMI: Shengmai injection
CHF: Chronic heart failure
RCTs: Randomized controlled trials
TCM: Traditional Chinese medicine
ACEIs: Angiotensin-converting enzyme inhibitors
LVEF: Left ventricular ejection fractions
NYHA: New York Heart Association
BNP: Brain natriuretic peptide
6MWT: Six-minute walk test
TNF-*α*: Tumor necrosis factor-*α*.

## Supplementary Materials

Supplementary Materials Figures S1–S12: plots of subgroup analyses. Table S1: sensitivity analysis excluding trials with a high risk of bias. Figures S13 – 17: funnel plot of publication bias.

## References

[1] Yancy C. W., Jessup M., Bozkurt B. (2013). ACCF/AHA guideline for the management of heart failure: a report of the american college of cardiology foundation/American heart association task force on practice guidelines. *Journal of the American College of Cardiology*.

[2] McMurray J. J., Adamopoulos S., Anker S. D. (2012). ESC guidelines for the diagnosis and treatment of acute and chronic heart failure 2012: the task force for the diagnosis and treatment of acute and chronic heart failure 2012 of the european society of cardiology. Developed in collaboration with the heart failure association (HFA) of the ESC. *European Heart Journal*.

[3] Orso F., Fabbri G., Maggioni A. P. (2017). Epidemiology of heart failure. *Handbook of experimental pharmacology*.

[4] Ambrosy A. P., Fonarow G. C., Butler J. (2014). The global health and economic burden of hospitalizations for heart failure. *Journal of the American College of Cardiology*.

[5] Yasuhiko S., Hiroaki S. (2013). Epidemiology of heart failure in Asia. *Circulation Journal:Official Journal of the Japanese Circulation Society*.

[6] Stout K. K., Broberg C. S., Book W. M. (2016). Chronic heart failure in congenital heart disease. *Circulation*.

[7] Ponikowski P., Voors A. A., Anker S. D. (2016). 2016 ESC guidelines for the diagnosis and treatment of acute and chronic heart failure. *Revista Española de Cardiología (English Edition)*.

[8] Ponikowski P., Voors A. A., Anker S. D. (2016). 2016 ESC guidelines for the diagnosis and treatment of acute and chronic heart failure. *Revista espanola de cardiologia*.

[9] Ponikowski P., Voors A. A., Anker S. D. (2016). ESC Guidelines for the diagnosis and treatment of acute and chronic heart failure: the task force for the diagnosis and treatment of acute and chronic heart failure of the european society of cardiology (ESC). developed with the special contribution of the heart failure association (HFA) of the ESC. *European Journal of Heart Failure*.

[10] Edelmann F., Knosalla C., Mörike K., Muth C., Prien P., Störk S. (2018). Chronic heart failure. *Deutsches Arzteblatt International*.

[11] Wang K., Zhang D., Wu J. (2017). A comparative study of Danhong injection and Salvia miltiorrhiza injection in the treatment of cerebral infarction: a systematic review and meta-analysis. *Medicine*.

[12] Feng Q. T., Yang J., Zhang Y., Lv S. Z., Miao H. W. (2019). Protective effect of buyiqiangxin tablet on chronic heart failure rats. *Proprietary Chinese Medicine*.

[13] Zhang Y., LI H., Wang C. X. (2011). Discussion on TCM disease name and pathogenesis of chronic heart failure. *Chinese Medicine*.

[14] Wang K. H., Wu J. R., Zhang D., Duan X. J., Ni M. W. (2018). Comparative efficacy of Chinese herbal injections for treating chronic heart failure: a network meta-analysis. *BMC Complementary and Alternative Medicine*.

[15] Zhan S. Y. (2014). *Study on Proteomics and in Vivo Process of Pharmacodynamic Substances in Shengmai Injection*.

[16] Deng Z. J. (2011). *Formulas of Chinese Medicine*.

[17] Zhang C. Q., huang M. Y., Cai X. J. (2020). Progress in clinical application of shengmai San. *Journal of Practical Traditional Chinese Medicine*.

[18] Zhang X. P., Chen Q. W. (2007). Effects of shengmai injection on ventricular remodeling in chronic heart failure rats. *Pharmacology and Clinic of Traditional Chinese Medicine*.

[19] Zhang X. M., Liu Y. (2013). Pharmacological mechanism and clinical application of shengmai injection. *Medical Review*.

[20] Peng C., Zhao G. (2012). Randomized controlled clinical study of shengmai injection combined with levocarnitine in the treatment of chronic heart failure. *Journal of Practical Traditional Chinese Internal Medicine*.

[21] Shen Q. B. (2018). Clinical effect of shengmai injection combined with cyclic adenosine on chronic heart failure and its effect on cardiac function. *Guangzhou Medicine*.

[22] Lai M. Q., Huang F. X., He Z. W., Wang L. J. (2018). Observation on the effect of shengmai injection in treating chronic heart failure. *Journal of Chronic Diseases*.

[23] Ding J. R., Zhou Y. W., Chen Y., Xiao X. Z. (2012). Effect of shengmai injection on cardiac function in elderly patients with chronic heart failure due to coronary heart disease. *International Journal of Geriatrics*.

[24] Li T. Q., Liu X. M., Feng M. (2009). Systematic review on the application and adverse reactions of shengmai injection. *Journal of Integrated Traditional and Western Medicine*.

[25] Liberati A., Altman D. G., Tetzlaff J. (2009). The PRISMA statement for reporting systematic reviews and meta-analyses of studies that evaluate health care interventions: explanation and elaboration. *Journal of Clinical Epidemiology*.

[26] McKee P. A., Castelli W. P., McNamara P. M., Kannel W. B. (1971). The natural history of congestive heart failure: the framingham study. *New England Journal of Medicine*.

[27] Wang C. (1994). Nomenclature and criteria for diagnosis of diseases of the heart. *Journal of the American Medical Association*.

[28] Higgins JPT G. S. (2011). Cochrane handbook for systematic reviews of interventions version 5.1.0. http://handbook.cochrane.

[29] Chen H., Wang Y. (2010). Observation on the effect of shengmai injection on chronic heart failure. *Aerospace Medicine*.

[30] Chi Y. S. (2017). Observation on the effect of shengmai injection combined with valsartan in the treatment of chronic heart failure. *Chinese Rural Medicine*.

[31] Dou C. J., Dou W., Zhang W. (2010). Observation on the curative effect of shengmai injection in treating chronic heart failure. *Journal of Gansu University of Traditional Chinese Medicine*.

[32] Kong L. G., Zhu K. W. (2004). Clinical observation of 30 cases of congestive heart failure treated by shengmai injection. *Journal of Clinical Emergency Call*.

[33] Ni Y. M. (2012). Shengmai injection assisted treatment of 43 cases of chronic heart failure. *The Medicine Herald*.

[34] Pan A. Q. (2013). Clinical study of shengmai injection combined with phentolamine in the treatment of chronic heart failure. *Practical Pharmacy And Clinical Remedies*.

[35] Pan J. J. (2014). Discussion on the therapeutic effect of shengmai injection on chronic heart failure. *Contemporary Medical*.

[36] Shi J. H. (2001). Observation on the effect of shengmai injection on chronic congestive heart failure. *Chinese Patent Medicine*.

[37] Sun G. R., Jing K. L., Quan Y. X. (2013). Effects of shengmai injection on cardiac function and heart rate variability in patients with slow heart attack. *Western Chinese Medicine*.

[38] Wang Y. X. (2010). Clinical observation of shengmai injection in the treatment of chronic congestive heart failure in the elderly. *Chinese Patent Medicine*.

[39] Wen Y. (2013). Observation on the effect of shengmai injection combined with western medicine in treating 64 cases of chronic heart failure. *Journal of Chengdu University of Traditional Chinese Medicine*.

[40] Wu J. B. (2014). Clinical observation on the treatment of qi Yin deficiency heart failure with shengmai injection. *Contemporary Medical*.

[41] Wu X. (2013). Clinical observation of 52 cases of chronic heart failure treated by shengmai injection. *Everyone Healthy*.

[42] Xu J. (2004). Shengmai injection assisted treatment of 50 cases of chronic heart failure. *Journal of Mudanjiang Medical College*.

[43] Ye L., Zhao L. L. (2011). Observation on the effect of shengmai injection on chronic congestive heart failure. *Shanxi Traditional Chinese Medicine*.

[44] Yuan Y. C. (2011). Clinical observation of 89 cases of coronary heart failure treated by shengmai injection combined with shuxuening. *Shandong Medicine*.

[45] Zhai Y. M. (2009). Clinical study of shengmai injection in treating 30 cases of chronic congestive heart failure. *Journal of Henan College of Traditional Chinese Medicine*.

[46] Zou X., Shi S. Q., Han Y. (2011). Clinical observation on the treatment of chronic heart failure with shengmai injection. *Clinical Journal of Traditional Chinese Medicine*.

[47] (2002). “ATS statement: guidelines for the six-minute walk test. *American Journal of Respiratory and Critical Care Medicine*.

[48] Liu X., Tan W., Yang F. (2018). Shengmai injection reduces apoptosis and enhances angiogenesis after myocardial ischaemia and reperfusion injury in rats. *Biomedicine & Pharmacotherapy*.

[49] Duckelmann C., Mittermayer F., Haider D. G. (2007). Asymmetric dimethylarginine enhances cardiovascular risk prediction in patients with chronic heart failure. *Arteriosclerosis, Thrombosis, and Vascular Biology*.

[50] Mao J. Y., Wang H. L., Wang Q. (2003). Effect of shengmai injection on serum TNF-a level in patients with heart failure. *New traditional Chinese medicine*.

[51] Chen C. Y., Lu L. Y., Chen P. (2013). Shengmai injection, a traditional Chinese patent medicine, for intradialytic hypotension: a systematic review and meta-analysis. *Evidence-based Complementary and Alternative Medicine*.

[52] Guo G. X., Wang J. X. (2015). Effects of shengmai injection on angiotensin ii, brain natriuretic peptide and ventricular remodeling in patients with chronic heart failure. *Pharmacology and Clinic of Traditional Chinese Medicine*.

[53] Cheng M., Jiang C. H., Huang P. (2011). Analysis of adverse reactions/events of shengmai injection in 1012 cases. *Anhui Medicine*.

