# Supplementary material for: Efficacy and Safety of Shengmai Injection for Chronic Heart Failure: A Systematic Review of Randomized Controlled Trials
Source: Evid Based Complement Alternat Med. 2020 Jun 20;2020:9571627. doi: 10.1155/2020/9571627 (PMC7322585; doi:10.1155/2020/9571627)
Supplement: Supplementary Materials — Figures S1–S12: plots of subgroup analyses. Table S1: sensitivity analysis excluding trials with a high risk of bias. Figures S13 – 17: funnel plot of publication bias. [file 9571627.f1.pdf]

PRISMA 2009 Checklist

Section/topic	#	Checklist item	Reported on page #
**TITLE**			
Title	1	Identify the report as a systematic review, meta-analysis, or both.	1
**ABSTRACT**			
Structured summary	2	Provide a structured summary including, as applicable: background; objectives; data sources; study eligibility criteria, participants, and interventions; study appraisal and synthesis methods; results; limitations; conclusions and implications of key findings; systematic review registration number.	3-4
**INTRODUCTION**			
Rationale	3	Describe the rationale for the review in the context of what is already known.	5-6
Objectives	4	Provide an explicit statement of questions being addressed with reference to participants, interventions, comparisons, outcomes, and study design (PICOS).	6
**METHODS**			
Protocol and registration	5	Indicate if a review protocol exists, if and where it can be accessed (e.g., Web address), and, if available, provide registration information including registration number.	N/A
Eligibility criteria	6	Specify study characteristics (e.g., PICOS, length of follow-up) and report characteristics (e.g., years considered, language, publication status) used as criteria for eligibility, giving rationale.	7
Information sources	7	Describe all information sources (e.g., databases with dates of coverage, contact with study authors to identify additional studies) in the search and date last searched.	6-7
Search	8	Present full electronic search strategy for at least one database, including any limits used, such that it could be repeated.	6-7
Study selection	9	State the process for selecting studies (i.e., screening, eligibility, included in systematic review, and, if applicable, included in the meta-analysis).	8
Data collection process	10	Describe method of data extraction from reports (e.g., piloted forms, independently, in duplicate) and any processes for obtaining and confirming data from investigators.	8-9
Data items	11	List and define all variables for which data were sought (e.g., PICOS, funding sources) and any assumptions and simplifications made.	8-9
Risk of bias in individual studies	12	Describe methods used for assessing risk of bias of individual studies (including specification of whether this was done at the study or outcome level), and how this information is to be used in any data synthesis.	8
Summary measures	13	State the principal summary measures (e.g., risk ratio, difference in means).	8-9
Synthesis of results	14	Describe the methods of handling data and combining results of studies, if done, including measures of consistency (e.g., I^2) for each meta-analysis.	8-9

PRISMA 2009 Checklist

Section/topic	#	Checklist item	Reported on page #
Risk of bias across studies	15	Specify any assessment of risk of bias that may affect the cumulative evidence (e.g., publication bias, selective reporting within studies).	9
Additional analyses	16	Describe methods of additional analyses (e.g., sensitivity or subgroup analyses, meta-regression), if done, indicating which were pre-specified.	9
**RESULTS**			
Study selection	17	Give numbers of studies screened, assessed for eligibility, and included in the review, with reasons for exclusions at each stage, ideally with a flow diagram.	9,Figure 1
Study characteristics	18	For each study, present characteristics for which data were extracted (e.g., study size, PICOS, follow-up period) and provide the citations.	9-11,Table 1
Risk of bias within studies	19	Present data on risk of bias of each study and, if available, any outcome level assessment (see item 12).	12,Figure 2
Results of individual studies	20	For all outcomes considered (benefits or harms), present, for each study: (a) simple summary data for each intervention group (b) effect estimates and confidence intervals, ideally with a forest plot.	12-15, Figure 3-7
Synthesis of results	21	Present results of each meta-analysis done, including confidence intervals and measures of consistency.	15-16, Figure 3-7
Risk of bias across studies	22	Present results of any assessment of risk of bias across studies (see Item 15).	16
Additional analysis	23	Give results of additional analyses, if done (e.g., sensitivity or subgroup analyses, meta-regression [see Item 16]).	16
**DISCUSSION**			
Summary of evidence	24	Summarize the main findings including the strength of evidence for each main outcome; consider their relevance to key groups (e.g., healthcare providers, users, and policy makers).	16-17
Limitations	25	Discuss limitations at study and outcome level (e.g., risk of bias), and at review-level (e.g., incomplete retrieval of identified research, reporting bias).	19
Conclusions	26	Provide a general interpretation of the results in the context of other evidence, and implications for future research.	19-20
**FUNDING**			
Funding	27	Describe sources of funding for the systematic review and other support (e.g., supply of data); role of funders for the systematic review.	20-21

From: Moher D, Liberati A, Tetzlaff J, Altman DG, The PRISMA Group (2009). Preferred Reporting Items for Systematic Reviews and Meta-Analyses: The PRISMA Statement. PLoS Med 6(7): e1000097. doi:10.1371/journal.pmed1000097

Supplementary Materials 1: Plots of subgroup analyses

	Shengmai injection			Basic treatment				Mean Difference
Study or Subgroup	Mean	SD	Total	Mean	SD	Total	Weight	IV, Random, 95% CI
**3.2.1 ≥ 60 ml**								
Chen 2010	25	6.557439	50	11	5.567764	50	5.7%	14.00 [11.62, 16.38]
Dou 2010	10.7	6.221736	30	5.6	6.062178	23	5.3%	5.10 [1.77, 8.43]
Lai 2018	4.28	2.608237	65	2.21	2.590926	65	6.0%	2.07 [1.18, 2.96]
Ni 2012	7.58	5.467111	43	5.92	5.498036	41	5.7%	1.66 [-0.69, 4.01]
Pan 2013	12.44	4.809626	34	7.05	4.263942	34	5.7%	5.39 [3.23, 7.55]
Sun 2013	19.1	4.70319	30	9.9	4.32088	30	5.7%	9.20 [6.91, 11.49]
Wu 2013	11.29	6.40353	30	3.51	6.290564	22	5.2%	7.78 [4.29, 11.27]
Xu 2004	8.9	7.312318	50	4.6	7.053368	26	5.3%	4.30 [0.91, 7.69]
**Subtotal (95% CI)**			**332**			**291**	**44.6%**	**6.16 [3.03, 9.29]**
Heterogeneity: Tau² = 18.61; Chi² = 115.04, df = 7 (P < 0.00001); I² = 94%								
Test for overall effect: Z = 3.86 (P = 0.0001)								
**3.2.2 < 60 ml**								
Chi 2017	19.4	4.687217	40	13.4	4.553021	40	5.8%	6.00 [3.97, 8.03]
Ding 2012	7.54	3.834697	40	4.79	4.2265	40	5.8%	2.75 [0.98, 4.52]
Kong 2004	20	4.582576	30	6	4.582576	30	5.7%	14.00 [11.68, 16.32]
Wang 2010	22	7	41	12	6.082763	41	5.5%	10.00 [7.16, 12.84]
Wen 2013	27	5.567764	32	9	6.928203	32	5.4%	18.00 [14.92, 21.08]
Wu 2014	11.1	5.311309	30	6.6	4.948737	30	5.6%	4.50 [1.90, 7.10]
Ye 2011	12	8.544004	30	8	9.539392	30	4.7%	4.00 [-0.58, 8.58]
Yuan 2011	5.31	5.286388	89	1.47	5.829691	89	5.9%	3.84 [2.21, 5.47]
Zhai 2009	21	4.358899	30	19.49	3.464102	30	5.8%	1.51 [-0.48, 3.50]
Zou 2011	18	8.544004	30	9	4.582576	30	5.2%	9.00 [5.53, 12.47]
**Subtotal (95% CI)**			**392**			**392**	**55.4%**	**7.32 [4.21, 10.43]**
Heterogeneity: Tau² = 23.26; Chi² = 155.66, df = 9 (P < 0.00001); I² = 94%								
Test for overall effect: Z = 4.61 (P < 0.00001)								
**Total (95% CI)**			**724**			**683**	**100.0%**	**6.80 [4.68, 8.91]**
Heterogeneity: Tau² = 19.13; Chi² = 283.86, df = 17 (P < 0.00001); I² = 94%								
Test for overall effect: Z = 6.29 (P < 0.00001)								
Test for subgroup differences: Chi² = 0.26, df = 1 (P = 0.61), I² = 0%								

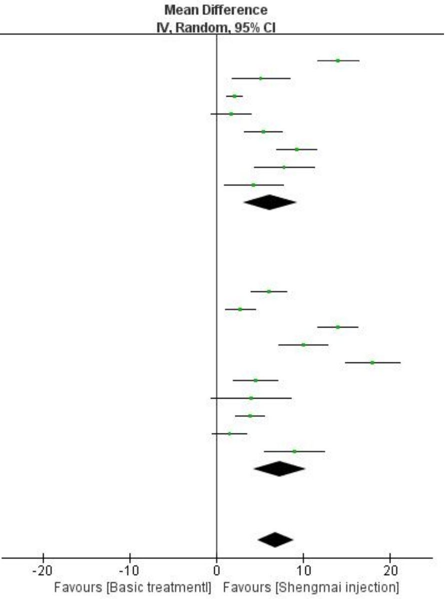

Figure S1. Subgroup analysis stratified by different doses of Shengmai injection for changes in left ventricular ejection fractions (%)

	Shengmai injection			Basic treatment				Mean Difference
Study or Subgroup	Mean	SD	Total	Mean	SD	Total	Weight	IV, Random, 95% CI
**4.2.1 ≥ 65 years**								
Ding 2012	7.54	3.834697	40	4.79	4.2265	40	5.8%	2.75 [0.98, 4.52]
Dou 2010	10.7	6.221736	30	5.6	6.062178	23	5.3%	5.10 [1.77, 8.43]
Zou 2011	18	8.544004	30	9	4.582576	30	5.2%	9.00 [5.53, 12.47]
**Subtotal (95% CI)**			**100**			**93**	**16.4%**	**5.39 [1.72, 9.07]**
Heterogeneity: Tau² = 8.38; Chi² = 10.21, df = 2 (P = 0.006); I² = 80%								
Test for overall effect: Z = 2.88 (P = 0.004)								
**4.2.2 < 65 years**								
Chen 2010	25	6.557439	50	11	5.567764	50	5.7%	14.00 [11.62, 16.38]
Chi 2017	19.4	4.687217	40	13.4	4.553021	40	5.8%	6.00 [3.97, 8.03]
Kong 2004	20	4.582576	30	6	4.582576	30	5.7%	14.00 [11.68, 16.32]
Lai 2018	4.28	2.608237	65	2.21	2.590926	65	6.0%	2.07 [1.18, 2.96]
Ni 2012	7.58	5.467111	43	5.92	5.498036	41	5.7%	1.66 [-0.69, 4.01]
Pan 2013	12.44	4.809626	34	7.05	4.263942	34	5.7%	5.39 [3.23, 7.55]
Sun 2013	19.1	4.70319	30	9.9	4.32088	30	5.7%	9.20 [6.91, 11.49]
Wang 2010	22	7	41	12	6.082763	41	5.5%	10.00 [7.16, 12.84]
Wen 2013	27	5.567764	32	9	6.928203	32	5.4%	18.00 [14.92, 21.08]
Wu 2013	11.29	6.40353	30	3.51	6.290564	22	5.2%	7.78 [4.29, 11.27]
Wu 2014	11.1	5.311309	30	6.6	4.948737	30	5.6%	4.50 [1.90, 7.10]
Xu 2004	8.9	7.312318	50	4.6	7.053368	26	5.3%	4.30 [0.91, 7.69]
Ye 2011	12	8.544004	30	8	9.539392	30	4.7%	4.00 [-0.58, 8.58]
Yuan 2011	5.31	5.286388	89	1.47	5.829691	89	5.9%	3.84 [2.21, 5.47]
Zhai 2009	21	4.358899	30	19.49	3.464102	30	5.8%	1.51 [-0.48, 3.50]
**Subtotal (95% CI)**			**624**			**590**	**83.6%**	**7.05 [4.60, 9.50]**
Heterogeneity: Tau² = 21.67; Chi² = 271.99, df = 14 (P < 0.00001); I² = 95%								
Test for overall effect: Z = 5.64 (P < 0.00001)								
**Total (95% CI)**			**724**			**683**	**100.0%**	**6.80 [4.68, 8.91]**
Heterogeneity: Tau² = 19.13; Chi² = 283.86, df = 17 (P < 0.00001); I² = 94%								
Test for overall effect: Z = 6.29 (P < 0.00001)								
Test for subgroup differences: Chi² = 0.54, df = 1 (P = 0.46), I² = 0%								

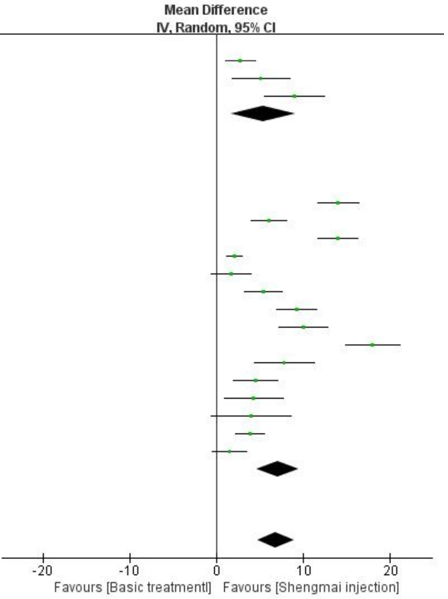

Figure S2. Subgroup analysis stratified by different years of Shengmai injection for changes in left ventricular ejection fractions (%)

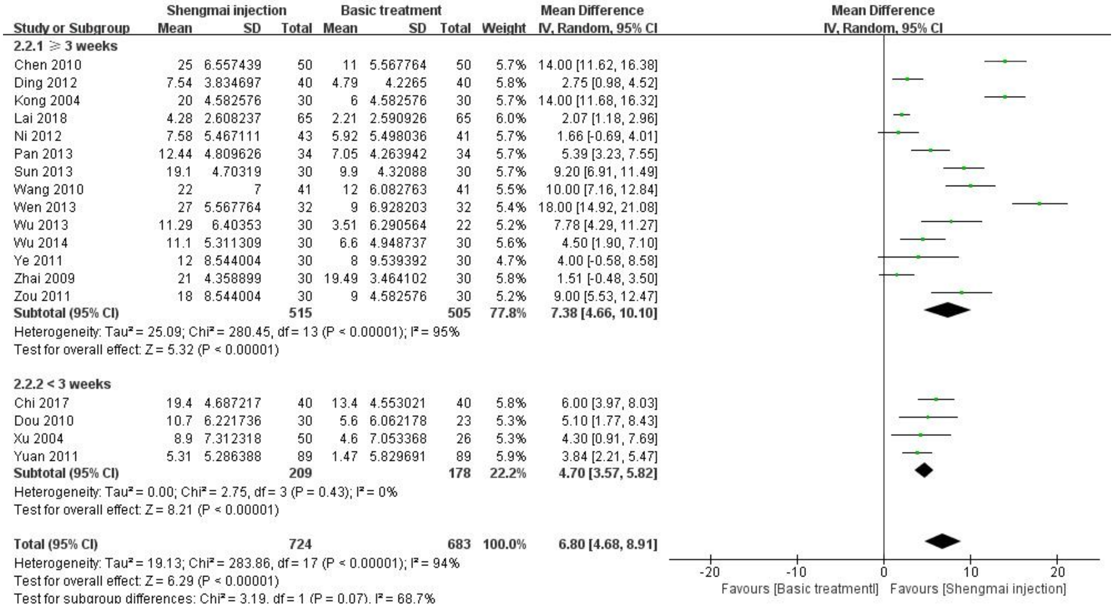

Figure S3. Subgroup analysis stratified by different follow-up of Shengmai injection for changes in left ventricular ejection fractions (%)

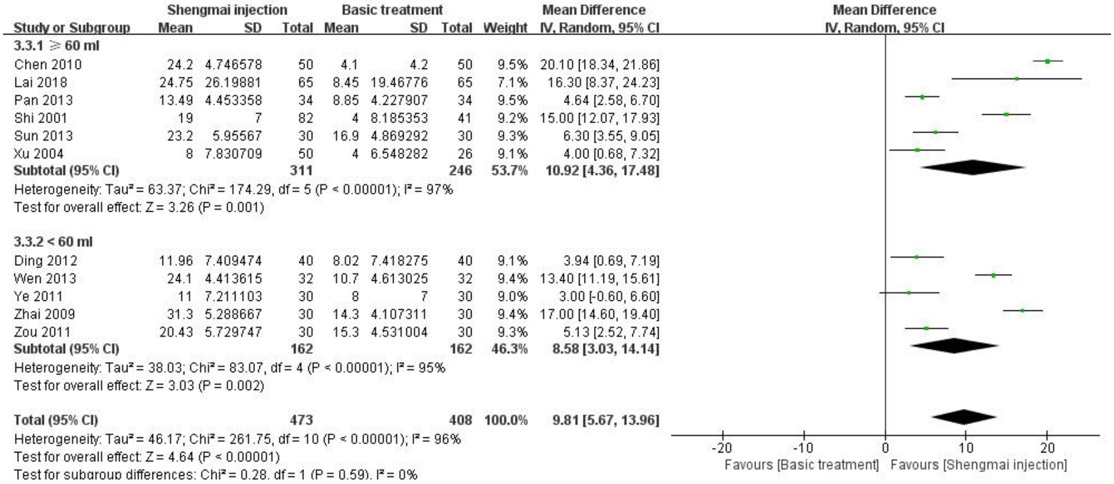

Figure S4. Subgroup analysis stratified different doses of Shengmai injection for changes in Stroke volume (ml)

Study or Subgroup	Shengmai injection Mean	SD	Total	Basic treatment Mean	SD	Total	Weight	Mean Difference IV, Random, 95% CI
**4.3.1 ≥ 65 years**								
Ding 2012	11.96	7.409474	40	8.02	7.418275	40	9.1%	3.94 [0.69, 7.19]
Shi 2001	19	7	82	4	8.185353	41	9.2%	15.00 [12.07, 17.93]
Zou 2011	20.43	5.729747	30	15.3	4.531004	30	9.3%	5.13 [2.52, 7.74]
**Subtotal (95% CI)**			**152**			**111**	**27.7%**	**8.03 [1.23, 14.84]**
Heterogeneity: Tau² = 33.92; Chi² = 32.58, df = 2 (P < 0.00001); I² = 94%								
Test for overall effect: Z = 2.31 (P = 0.02)								
**4.3.2 < 65 years**								
Chen 2010	24.2	4.746578	50	4.1	4.2	50	9.5%	20.10 [18.34, 21.86]
Lai 2018	24.75	26.19881	65	8.45	19.46776	65	7.1%	16.30 [8.37, 24.23]
Pan 2013	13.49	4.453358	34	8.85	4.227907	34	9.5%	4.64 [2.58, 6.70]
Sun 2013	23.2	5.95567	30	16.9	4.869292	30	9.3%	6.30 [3.55, 9.05]
Wen 2013	24.1	4.413615	32	10.7	4.613025	32	9.4%	13.40 [11.19, 15.61]
Xu 2004	8	7.830709	50	4	6.548282	26	9.1%	4.00 [0.68, 7.32]
Ye 2011	11	7.211103	30	8	7	30	9.0%	3.00 [-0.60, 6.60]
Zhai 2009	31.3	5.288667	30	14.3	4.107311	30	9.4%	17.00 [14.60, 19.40]
**Subtotal (95% CI)**			**321**			**297**	**72.3%**	**10.50 [5.38, 15.63]**
Heterogeneity: Tau² = 51.37; Chi² = 212.68, df = 7 (P < 0.00001); I² = 97%								
Test for overall effect: Z = 4.02 (P < 0.0001)								
**Total (95% CI)**			**473**			**408**	**100.0%**	**9.81 [5.67, 13.96]**
Heterogeneity: Tau² = 46.17; Chi² = 261.75, df = 10 (P < 0.00001); I² = 96%								
Test for overall effect: Z = 4.64 (P < 0.00001)								
Test for subgroup differences: Chi² = 0.32, df = 1 (P = 0.57), I² = 0%								

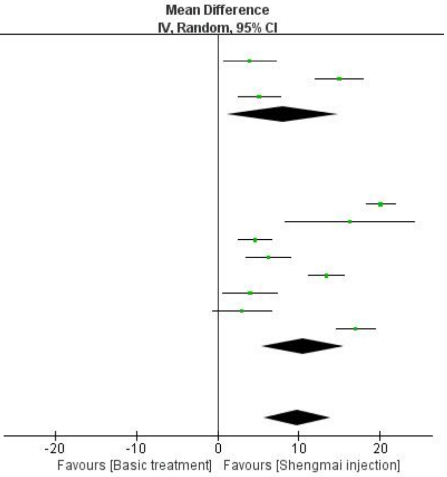

Figure S5. Subgroup analysis stratified by different years of Shengmai injection for changes in Stroke volume (ml)

Study or Subgroup	Shengmai injection Mean	SD	Total	Basic treatment Mean	SD	Total	Weight	Mean Difference IV, Random, 95% CI
**2.3.1 ≥ 3 weeks**								
Chen 2010	24.2	4.746578	50	4.1	4.2	50	9.5%	20.10 [18.34, 21.86]
Ding 2012	11.96	7.409474	40	8.02	7.418275	40	9.1%	3.94 [0.69, 7.19]
Lai 2018	24.75	26.19881	65	8.45	19.46776	65	7.1%	16.30 [8.37, 24.23]
Pan 2013	13.49	4.453358	34	8.85	4.227907	34	9.5%	4.64 [2.58, 6.70]
Shi 2001	19	7	82	4	8.185353	41	9.2%	15.00 [12.07, 17.93]
Sun 2013	23.2	5.95567	30	16.9	4.869292	30	9.3%	6.30 [3.55, 9.05]
Wen 2013	24.1	4.413615	32	10.7	4.613025	32	9.4%	13.40 [11.19, 15.61]
Ye 2011	11	7.211103	30	8	7	30	9.0%	3.00 [-0.60, 6.60]
Zhai 2009	31.3	5.288667	30	14.3	4.107311	30	9.4%	17.00 [14.60, 19.40]
Zou 2011	20.43	5.729747	30	15.3	4.531004	30	9.3%	5.13 [2.52, 7.74]
**Subtotal (95% CI)**			**423**			**382**	**90.9%**	**10.40 [6.05, 14.74]**
Heterogeneity: Tau² = 46.09; Chi² = 243.24, df = 9 (P < 0.00001); I² = 96%								
Test for overall effect: Z = 4.69 (P < 0.00001)								
**2.3.2 < 3 weeks**								
Xu 2004	8	7.830709	50	4	6.548282	26	9.1%	4.00 [0.68, 7.32]
**Subtotal (95% CI)**			**50**			**26**	**9.1%**	**4.00 [0.68, 7.32]**
Heterogeneity: Not applicable								
Test for overall effect: Z = 2.36 (P = 0.02)								
**Total (95% CI)**			**473**			**408**	**100.0%**	**9.81 [5.67, 13.96]**
Heterogeneity: Tau² = 46.17; Chi² = 261.75, df = 10 (P < 0.00001); I² = 96%								
Test for overall effect: Z = 4.64 (P < 0.00001)								
Test for subgroup differences: Chi² = 5.26, df = 1 (P = 0.02), I² = 81.0%								

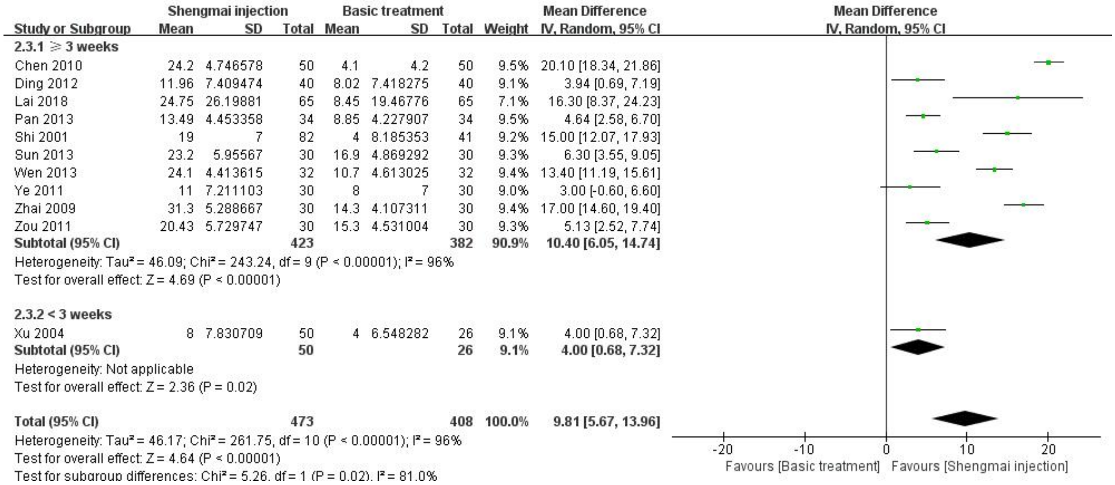

Figure S6. Subgroup analysis stratified by different follow-up of Shengmai injection for changes in Stroke volume (ml)

Study or Subgroup	Shengmai injection Mean	SD	Total	Basic treatment Mean	SD	Total	Weight	Mean Difference IV, Random, 95% CI
**3.4.1 ≥ 60 ml**								
Chen 2010	2.47	0.443058	50	1.71	0.4531	50	9.7%	0.76 [0.58, 0.94]
Lai 2018	2.34	1.993815	65	0.93	2.023191	65	6.5%	1.41 [0.72, 2.10]
Pan 2013	1.66	1.091375	34	0.64	1.008167	34	7.8%	1.02 [0.52, 1.52]
Shi 2001	1.63	1.167733	82	0.64	1.196161	41	8.2%	0.99 [0.55, 1.43]
Sun 2013	1.6	0.52915	30	0.4	0.43589	30	9.4%	1.20 [0.95, 1.45]
Xu 2004	0.67	0.393954	50	0.39	0.615061	26	9.3%	0.28 [0.02, 0.54]
**Subtotal (95% CI)**			**311**			**246**	**50.8%**	**0.90 [0.58, 1.22]**
Heterogeneity: Tau² = 0.12; Chi² = 30.14, df = 5 (P < 0.0001); I² = 83%								
Test for overall effect: Z = 5.48 (P < 0.00001)								
**3.4.2 < 60 ml**								
Ding 2012	1.27	0.72581	40	0.83	0.514684	40	9.2%	0.44 [0.16, 0.72]
Kong 2004	3	0.180278	30	1.3	0.353412	20	9.7%	1.70 [1.53, 1.87]
Wen 2013	2.76	0.381576	32	1.5	0.458258	32	9.5%	1.26 [1.05, 1.47]
Ye 2011	1.4	1.311488	30	0.7	1.352775	30	6.6%	0.70 [0.03, 1.37]
Zhai 2009	1.3	1.216553	30	0.5	1.479865	30	6.5%	0.80 [0.11, 1.49]
Zou 2011	1.5	0.953939	30	0.61	1.126943	30	7.6%	0.89 [0.36, 1.42]
**Subtotal (95% CI)**			**192**			**182**	**49.2%**	**0.99 [0.52, 1.46]**
Heterogeneity: Tau² = 0.29; Chi² = 66.57, df = 5 (P < 0.00001); I² = 92%								
Test for overall effect: Z = 4.15 (P < 0.0001)								
**Total (95% CI)**			**503**			**428**	**100.0%**	**0.96 [0.66, 1.25]**
Heterogeneity: Tau² = 0.23; Chi² = 128.94, df = 11 (P < 0.00001); I² = 91%								
Test for overall effect: Z = 6.27 (P < 0.00001)								
Test for subgroup differences: Chi² = 0.11, df = 1 (P = 0.74), I² = 0%								

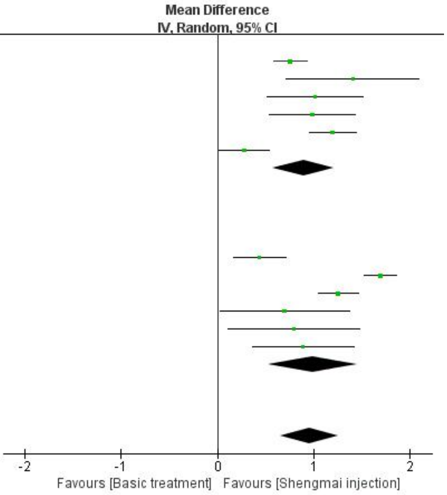

Figure S7. Subgroup analysis stratified different doses of Shengmai injection for changes in Cardiac output (l/min)

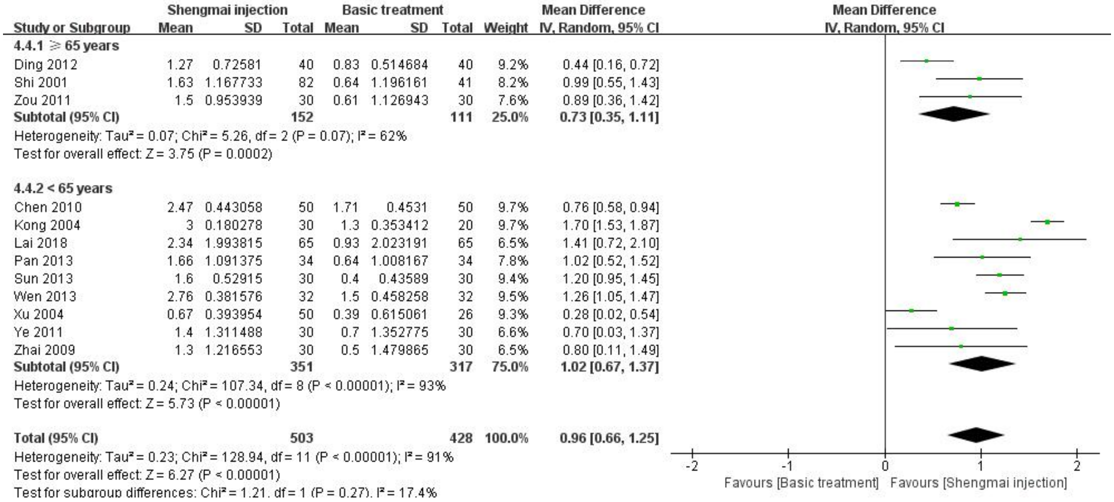

Figure S8. Subgroup analysis stratified by different years of Shengmai injection for changes in Cardiac output (l/min)

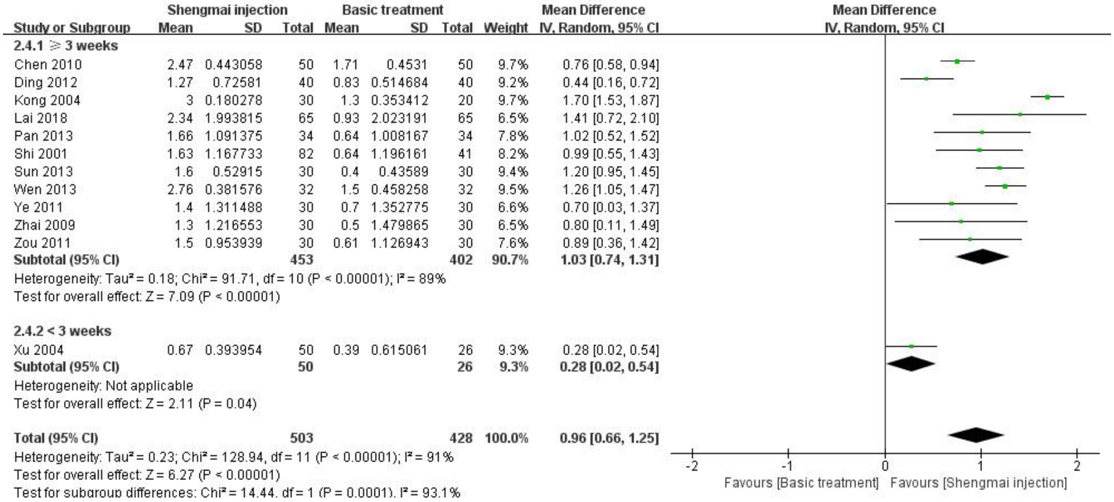

Figure S9. Subgroup analysis stratified by different follow-up of Shengmai injection for changes in Cardiac output (l/min)

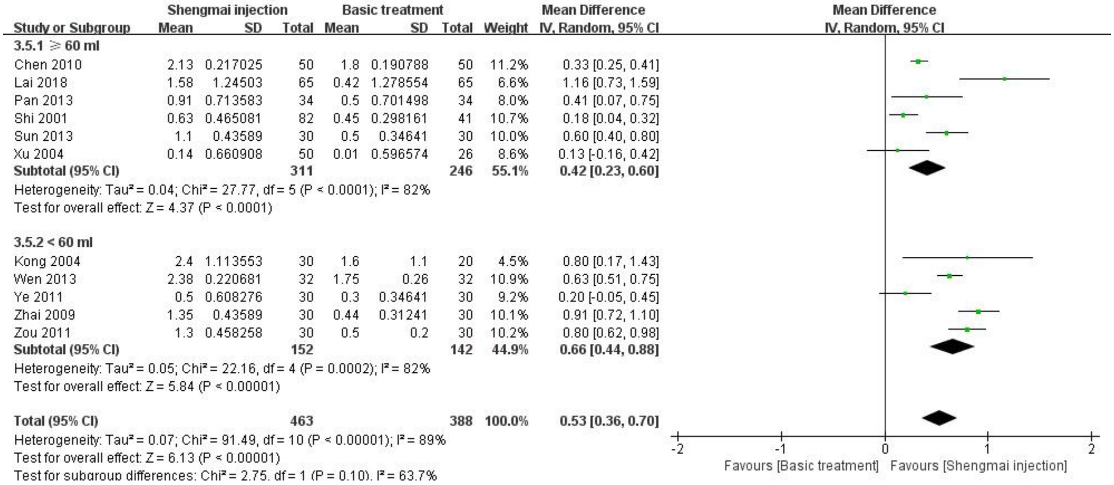

Figure S10. Subgroup analysis stratified different doses of Shengmai injection for changes in Cardiac index (l/min)

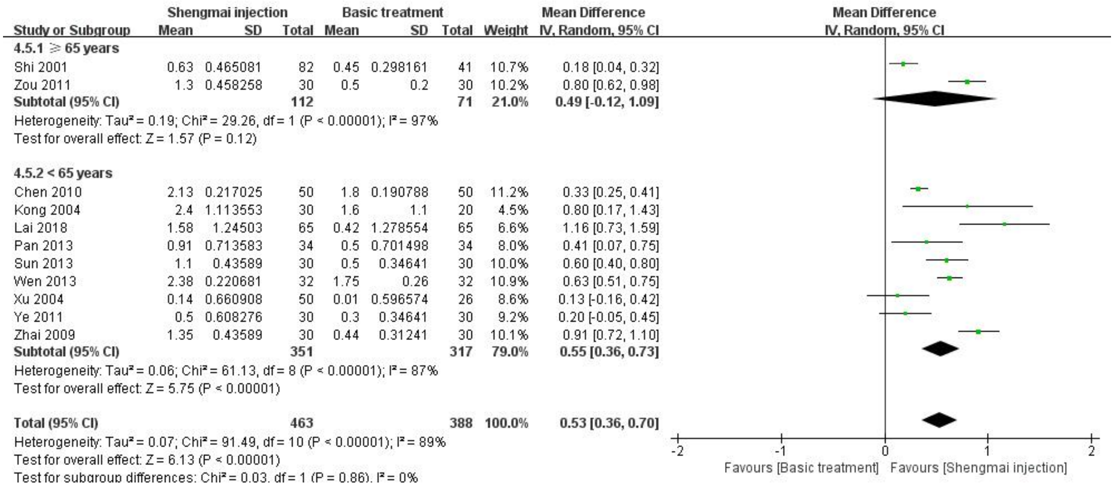

Figure S11. Subgroup analysis stratified by different years of Shengmai injection for changes in Cardiac index (l/min)

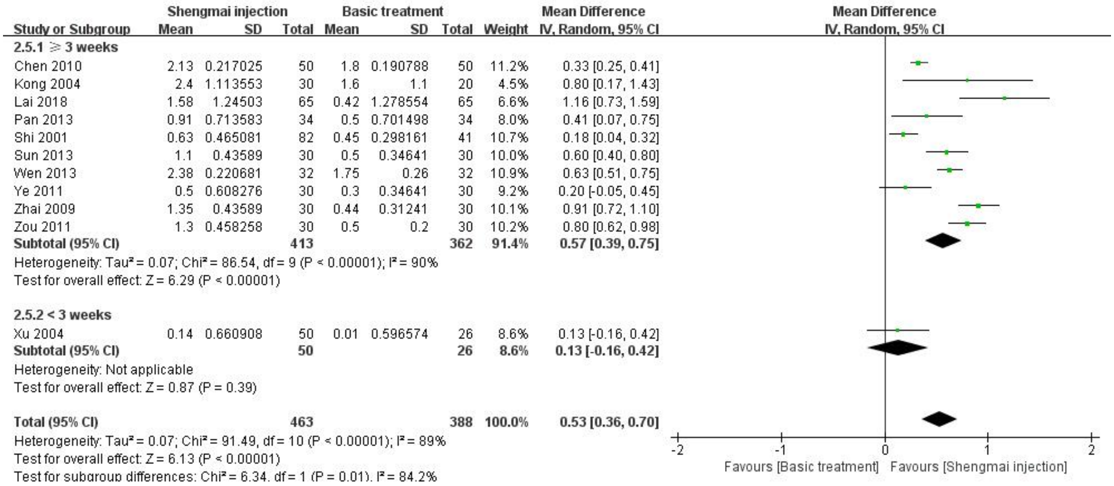

Figure S12. Subgroup analysis stratified by different follow-up of Shengmai injection for changes in Cardiac index (l/min)

Supplementary Materials 2: Results of sensitivity analysis

Table S1. Sensitivity analysis excluding trials with a high risk of bias

**Outcome**	**Analysis**	**No. of pts**		**Mean difference (95% CI)**	**Heterogeneity**
		**Shengmai**	**Control**		
left ventricular ejection fractions	Main analysis	724	683	6.80 (4.68, 8.91)	I^2=99%, P<0.0001
	Excluding trials with high risk of bias	624	583	6.85 (4.36, 9.34)	I^2=95%, P<0.0001
Response to treatment	Main analysis	662	595	2.89 (2.10, 3.99)	I^2=0%, P<0.0001
	Excluding trials with high risk of bias	562	495	2.77 (1.95, 3.92)	I^2=0%, P<0.0001
Stroke volume	Main analysis	473	408	9.81 (5.67, 13.96)	I^2=96%, P<0.00001
	Excluding trials with high risk of bias	443	378	10.18(5.71,14.64)	I^2=96%, P<0.00001
Cardiac output	Main analysis	503	428	0.96 (0.66, 1.25)	I^2=91%, P<0.00001
	Excluding trials with high risk of bias	473	398	0.93 (0.60, 1.26)	I^2=92%, P<0.00001
Cardiac index	Main analysis	463	388	0.53 (0.36, 0.70)	I^2=89%, P<0.00001
	Excluding trials with high risk of bias	433	358	0.53 (0.34, 0.71)	I^2=90%, P<0.00001
six-minute walk	Main analysis	125	118	70.67 (22.92,118.42)	I2=84%, P=0.004
	Excluding trials with high risk of bias	95	88	64.23 (-13.55,142.02)	I2=92%, P=0.11
Brain natriuretic peptide	Main analysis	197	195	-284.66 (-353.73, -215.59)	I^2=0%, P<0.00001
	Excluding trials with high risk of bias	197	195	-284.66 (-353.73, -215.59)	I^2=0%, P<0.00001

Supplementary Materials 3: Results of publication bias

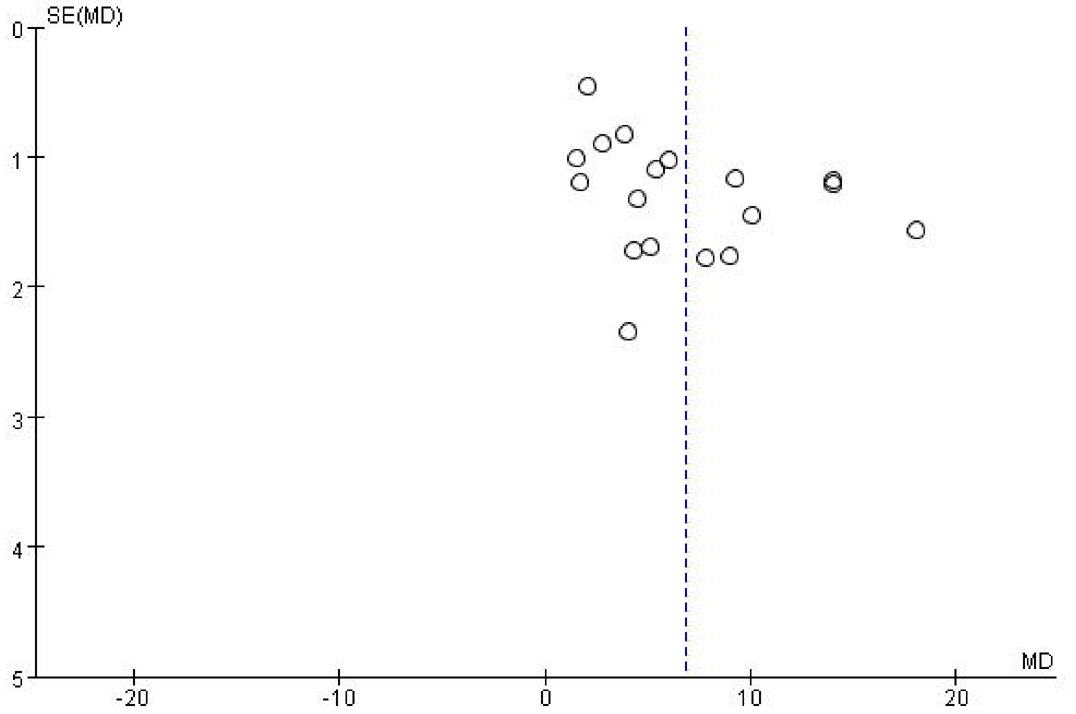

Figure S13. Funnel plot of left ventricular ejection fractions

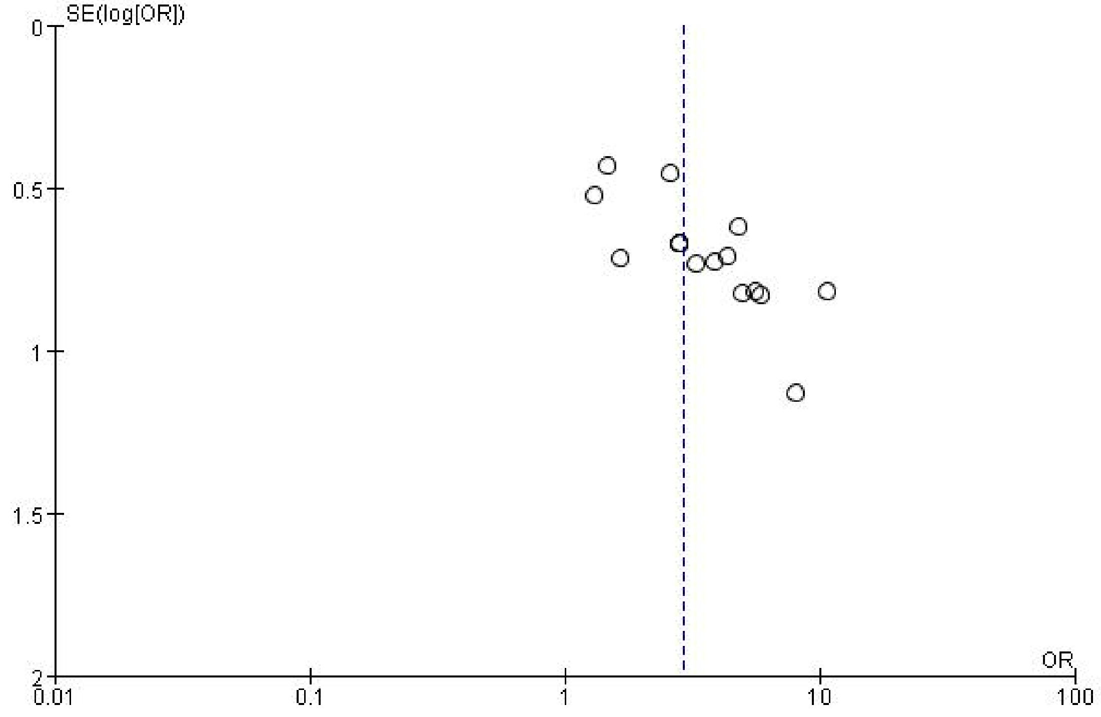

Figure S14. Funnel plot of response to

treatment

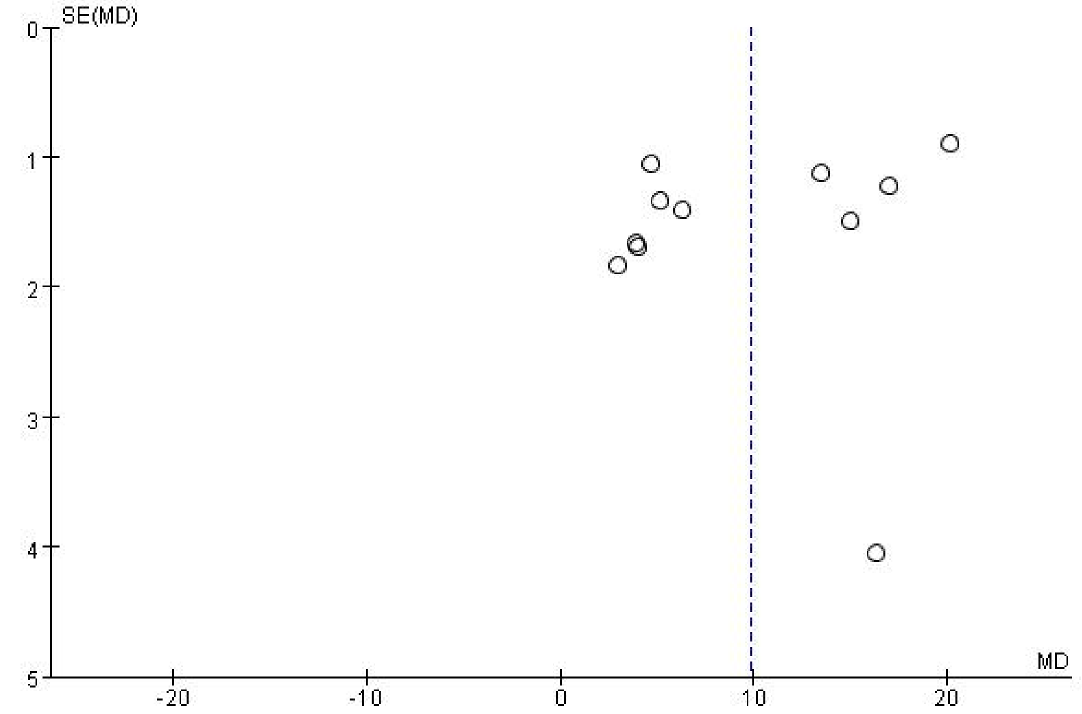

Figure S15. Funnel plot of stroke volume

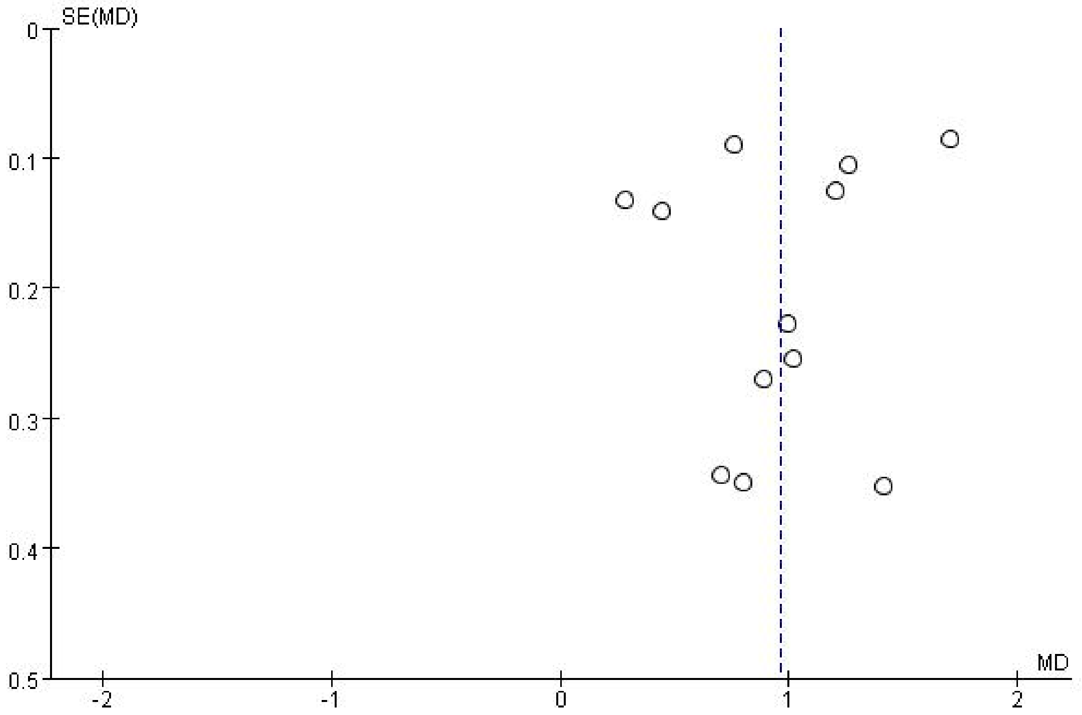

Figure S16. Funnel plot of cardiac output

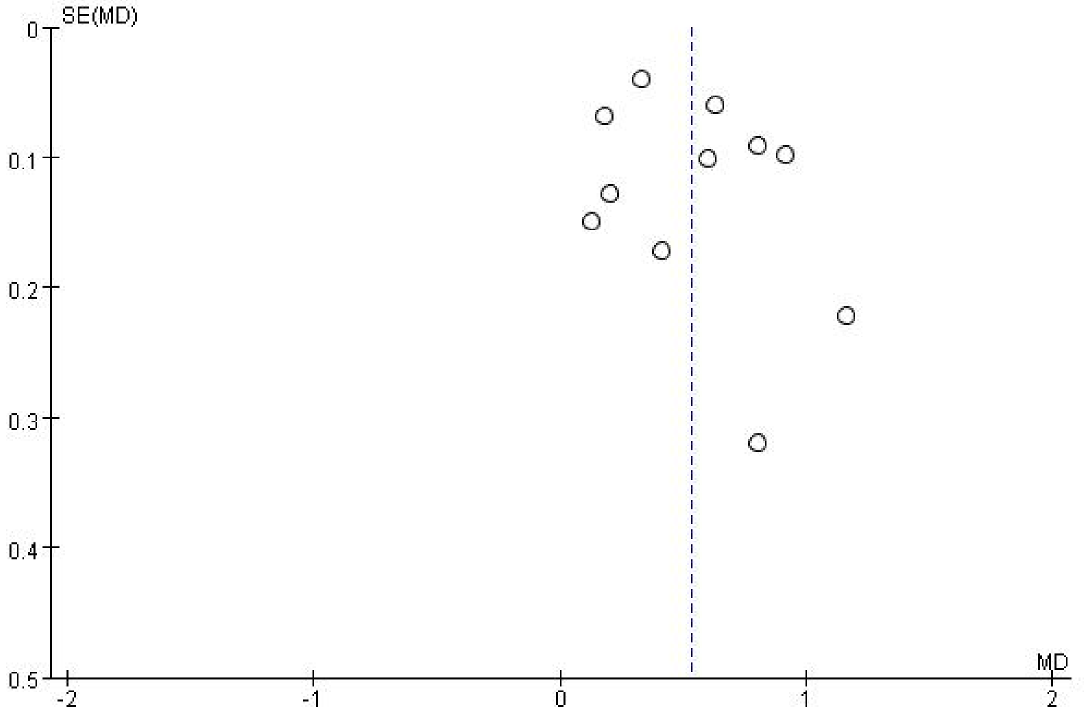

Figure S17. Funnel plot of cardiac index
